# Efficacy and safety of bulleyaconitine A in the treatment of osteoarthritis: A systematic review and meta-analysis

**DOI:** 10.1097/MD.0000000000044389

**Published:** 2025-09-12

**Authors:** Min Xiao, Xia-han Huang, Fang-lan Ma

**Affiliations:** a Department of Nephropathy Rheumatology Immunology, Xichang People’s Hospital, Xichang, Sichuan Province, China; b Department of Gastroenterology, Liangshan Hospital of Integrated Traditional Chinese and Western Medicine, Xichang, Sichuan Province, China.

**Keywords:** bulleyaconitine A, efficacy, meta-analysis, osteoarthritis, safety

## Abstract

**Background:**

This study evaluated the efficacy and safety of bulleyaconitine A in the treatment of osteoarthritis compared to non–bulleyaconitine A interventions.

**Methods:**

We searched PubMed, Embase, Web of Science, Cochrane Library, CBM, CNKI, VIP data, Wan Fang Data, ClinicalTrials.gov, the WHO International Clinical Trial Registry Platform (WHO ICTRP), and the Chinese Clinical Trial Registry (ChiCTR) from their inception to March 1, 2024, and selected relevant records based on inclusion criteria. Two independent reviewers screened the literature, extracted data, and assessed the risk of bias of the included studies. The meta-analysis was conducted using RevMan 5.3 software.

**Results:**

The study included 5 randomized controlled trials with a total of 424 participants, which revealed that the efficacy rate and Visual Analog Scale score of bulleyaconitine A for treating osteoarthritis were comparable to those of conventional therapy. However, the knee joint function improvement in the experimental group exceeded that of the control group, and there was a reduced incidence of adverse events.

**Conclusion:**

Bulleyaconitine A is effective in the treatment of osteoarthritis and has few adverse events.

## 1. Introduction

Osteoarthritis (OA) is the most common degenerative joint disorder, primarily affecting the knee, hip, spine, and hand joints. Its clinical manifestations include cartilage degeneration, bone remodeling, osteophyte formation, and synovitis,^[1]^ leading to pain, stiffness, swelling and functional impairment of the affected joint. The prevalence of OA is gradually increasing owing to the acceleration of global population aging, the rising proportion of obesity, and an increase in the incidence of joint trauma, with more than 400 million OA patients worldwide,^[2]^ making it the primary cause of disability among the elderly population. The burden of OA on individuals is significant. Globally, the average annual cost per person ranges from $700 to $15,600 (2019 USD).^[3]^ Additionally, 31% of OA patients have 5 or more comorbid conditions. OA patients also face a 20% higher mortality rate compared to age-matched controls, partly due to reduced physical activity.^[4,5]^ To address this growing burden, there is a need for more effective and safer treatments.

The purpose of OA treatment is to reduce pain, improve limb function, improve quality of life and reduce the incidence of disability, the current treatment modalities primarily include physical therapy, drug therapy, and surgical intervention. In the treatment of mild to moderate osteoarthritis, drug therapy is the most important means of treatment, and oral nonsteroidal anti-inflammatory drugs (NSAIDs) are the recommended first-line treatment according to guidelines. However, the gastrointestinal and cardiovascular adverse events associated with the prolonged use of NSAIDs pose challenges to both patients and medical practitioners, and alternative drugs for the treatment of OA have been a major focus of research.

The bulleyaconitine A (BLA) is a medicinal plant that exhibits potent analgesic and anti-inflammatory effects.^[6]^ In recent years, there has been an increasing utilization of BLA in the treatment of OA. The efficacy and safety of BLA currently lacks sufficient systematic reviews and analyses. The objective of this study was to conduct a systematic review of randomized controlled clinical trials (RCTs) investigating the efficacy of BLA, both as a standalone treatment and in combination with conventional drugs, for OA. This study aimed to provide robust evidence-based guidance for the treatment of OA and the clinical use of BLA.

## 2. Materials and methods

### 2.1. Study protocol

The analysis was conducted in accordance with a predetermined protocol, adhering to the recommendations outlined in the Cochrane Handbook of Systematic Reviews. The entire process was conducted strictly in accordance with the guidelines outlined in the Cochrane Handbook of Systematic Reviews and Meta-analysis of Interventions. The protocol was registered with INPLASY (No. 202430034).

### 2.2. Search strategy

Eleven databases were searched for relevant studies. The search was conducted across 4 Chinese databases (CBM, CNKI, VIP, and Wan Fang), 4 English databases (Pubmed, Embase, Web of Science, and Cochrane Library), and 3 clinical trial databases (ClinicalTrials.gov, WHO ICTRP, and ChiCTR) with a language restriction of English or Chinese. The key words for searching were “Osteoarthritis” and “Bulleyaconitine A.” The search time was from their inception to March 1, 2024. The detailed search strategies are provided in Appendix A, Supplemental Digital Content, https://links.lww.com/MD/P892.

### 2.3. Selection criteria

#### 2.3.1. Participants

Patients over 18 years of age were diagnosed with OA according to the recognized criteria. There were no restrictions on gender, age, ethnicity, etc.

#### 2.3.2. Intervention

The experimental group received BLA (tablets or capsules) or conventional therapies. The control group received different drugs or conventional therapy.

#### 2.3.3. Outcomes

The primary outcome measures included effective rate, VAS score, Lequesne score, Lysholm score, WOMAC score, and adverse events.

#### 2.3.4. Study design

RCTs, with no restrictions on publication time, quality or status.

### 2.4. Exclusion criteria

Duplicate publications. The full text was unavailable, crucial outcome measures were not accessible, or and data could not be converted. The participants were under 18 years of age. Non-Chinese and English literature.

### 2.5. Literature screening and data extraction

Two independent reviewers screened the literature, extracted and cross-checked the data. Any disagreements were resolved through discussions by all reviewers. The authors were contacted to compensate for the lack of relevant data. If the authors cannot be reached, try extracting data from relevant pictures. During literature screening, the title and abstract were initially reviewed, followed by a thorough examination of the full text to determine its inclusion in the final selection. The data extraction mainly includes: basic study information such as title, first author, publication journal, and time; baseline characteristics of the study subjects including region, number of cases, gender, age, inclusion and exclusion criteria, number of dropouts, etc; the specific details of intervention measures and follow-up time; key elements of risk assessment bias; Outcome indicators and measurement data of interest (including effective rate, VAS score, Lequesne score, Lysholm score, WOMAC score, Quantitative score of Traditional Chinese Medicine syndrome grading and adverse events).

### 2.6. Risk of bias assessment

Two reviewers independently evaluated the quality of the RCTs using the Cochrane risk-of-bias assessment tool. Disagreements were discussed by all the reviewers. This tool includes 6 aspects: random sequence generation, allocation concealment, blinding, incomplete outcomes, selective reporting, and other bias. Each item was recorded as having a low risk of bias, high risk of bias, or unclear risk of bias.

### 2.7. Statistical analysis

RevMan 5.3 software was used for statistical analysis. Binary data are expressed as relative risk and 95% confidence interval. For continuous variables, the standard MD was used as the effect size indicator when measurement units or values differed significantly. Otherwise, the mean difference (MD) was used. The chi-square test was used to evaluate heterogeneity. *P* > .1 and *I*^2^ < 50%, a fixed effect model was used for meta-analysis due to the lack of statistical heterogeneity among the studies. However, if *P* < .1 and *I*^2^ > 50%, statistical heterogeneity was present, requiring sensitivity analysis or subgroup analysis to identify the source of heterogeneity. Once the source was removed, tests were conducted to ensure consistency of the results. If the source of heterogeneity could not be determined, a random effects model was used for the meta-analysis.

## 3. Results

### 3.1. Results of the search

**Figure 1.**
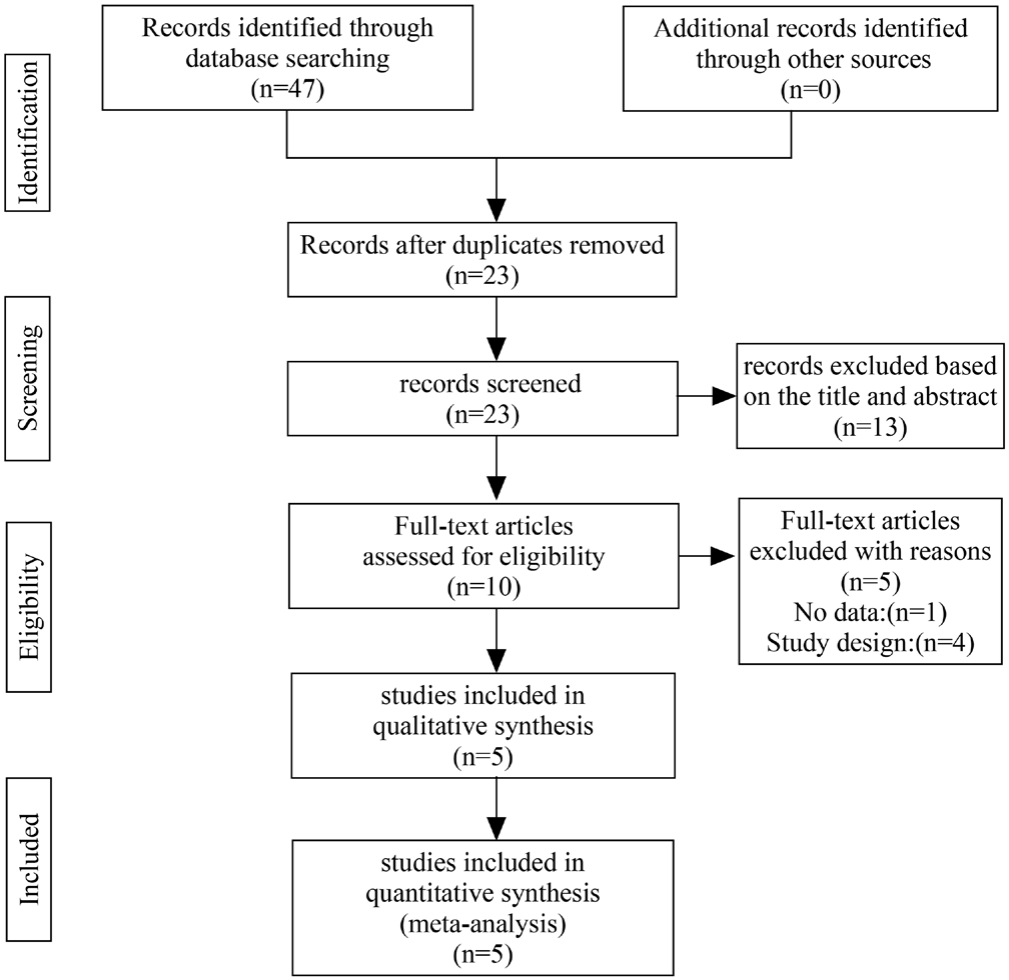
Flowchart of the articles search and screening process.

A total of 47 relevant articles were obtained from eleven databases. PubMed, 2; Embase, 3; Web of Science, 0; Cochrane Library, 0; CBM, 16; CNKI, 10; VIP, 7; Wan Fang, 9; ClinicalTrials.gov, 0; WHO ICTRP, 0; ChiCTR, 0. After removing duplicate articles, 23 articles remained; after reading titles and abstracts and eliminating irrelevant articles, only 10 articles were left. Finally, after excluding literature without experimental data and inconsistent control measures, 5 studies were obtained.^[7–11]^ The flow chart and results of the literature screening are shown in Figure 1.

### 3.2. Description of included trials

Five RCTS involving 424 patients were included in this study. Shen et al^[9]^ and Xu et al^[11]^ used NSAIDs as the comparator, Du et al^[7]^ and Xiao et al^[10]^ used traditional Chinese medicine, and Pan et al^[8]^ used hyaluronic acid as the comparator. The basic characteristics of the included studies are shown in Table 1.

**Table 1:** The characteristics of the included studies.

Study	Country	Sample size (female/male)		Intervention		
		Trial group	Control group	Trial group		Control group
Xu et al^[11]^	China	57 (50/7)	57 (46/11)	Bulleyaconitine A tablets 1.2 mg/d		Diclofenac 75 mg/d
Pan et al^[8]^	China	45 (29/16)	45 (30/15)	Bulleyaconitine A tablets 0.8 mg/d + Hyaluronic acid		Hyaluronic acid
Xiao et al^[10]^	China	50 (26/24)	50 (22/28)	Bulleyaconitine A capsule 1.2 g/d + Chinese Medicine Shuangbai San		Chinese Medicine Shuangbai San
Shen et al^[9]^	China	30 (22/8)	30 (23/7)	Bulleyaconitine A tablets 1.2 mg/d + Hyaluronic acid		Diclofenac 150 mg/d + Hyaluronic acid
Du et al^[7]^	China	30	30	Bulleyaconitine A tablets 1.2 mg/d + Tripterygium glycosides		Tripterygium glycosides

Study	Relevant outcomes	Mean age (yr)		Duration of illness (years or days)		Treatment duration (wk)
		Trial group	Control group	Trial group	Control group	
Xu et al^[11]^	VAS, WOMAC score, effective rate, adverse events	60 ± 9	56 ± 11	6 ± 5 (yr)	7 ± 5 (yr)	2
Pan et al^[8]^	VAS, lenquesne score, lysholm score, adverse events	60 ± 18	62 ± 17	45 ± 15 (d)	40 ± 21 (d)	2
Xiao et al^[10]^	VAS, WOMAC score, effective rate, adverse events	60.2 ± 8.56	59.5 ± 5.43	6.52 ± 1.54 (yr)	5.68 ± 2.36 (yr)	4
Shen et al^[9]^	Tegner score, effective rate, adverse events	68.8	72.3	2.3 (yr)	2.7 (yr)	2
Du et at^[7]^	VAS, quantitative score of TCM syndrome grading	51.43 ± 8.66	52.42 ± 7.18	2.54 ± 1.14 (yr)	2.38 ± 1.10 (yr)	4

TCM = traditional Chinese medicine, VAS = visual analogue scale, WOMAC = Western Ontario and McMaster Universities Osteoarthritis Index.

#### 3.2.1. Risk of bias of included studies

**Figure 2.**
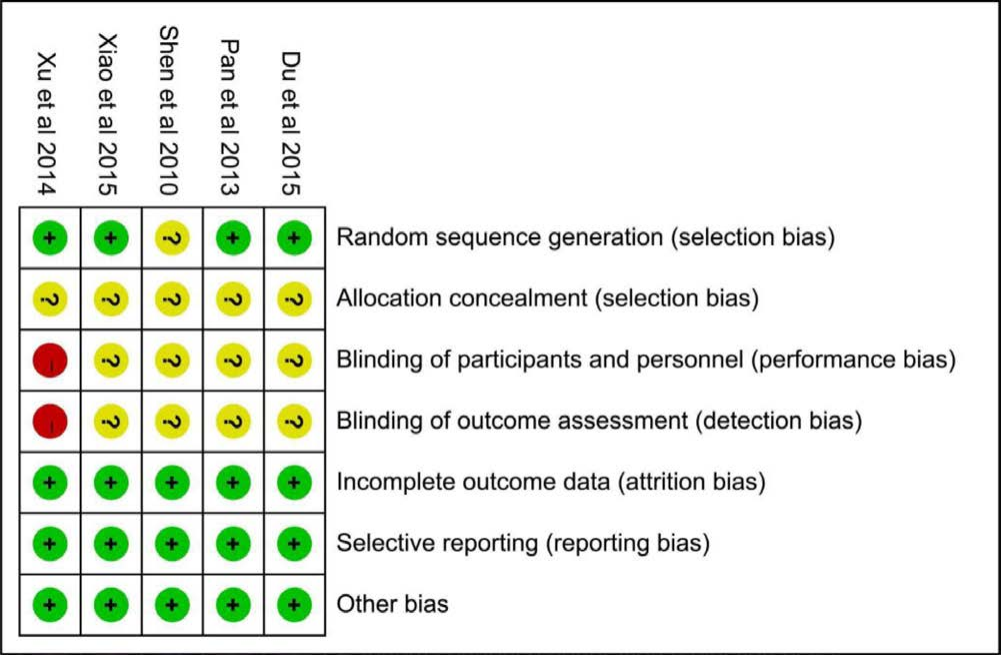
Risk of bias summary.

**Figure 3.**
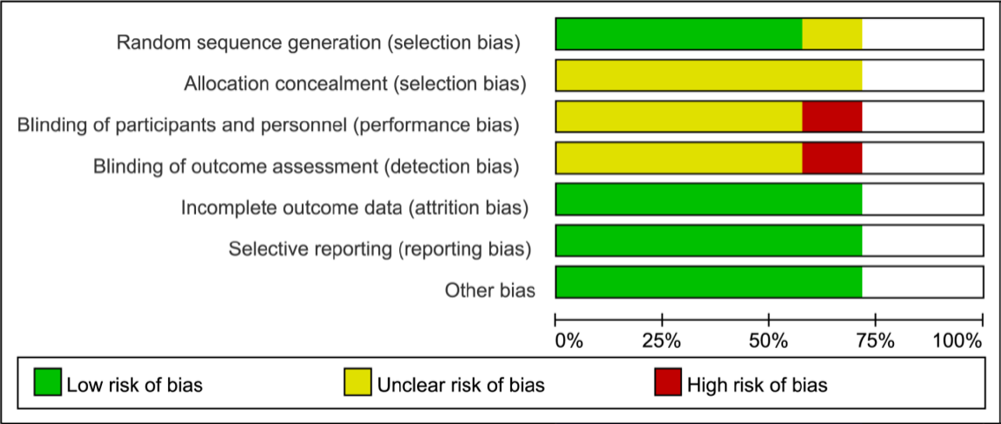
Risk of bias graph.

The Cochrane bias risk assessment tool was used by 2 researchers to conduct a rigorous quality assessment of the included literature. The summary and graph of the risk of bias are shown in Figures 2 and 3.

#### 3.2.2. Random sequence generation

Random sequence generation methods were not described in 1 RCT, resulting in an unclear risk of bias rating. The other 4 studies were rated as having a low risk of bias.

#### 3.2.3. Allocation concealment

None of the 5 RCT described whether allocation concealment was performed, therefore; they were assessed as having an unclear risk of bias.

#### 3.2.4. Blinding

One study was an open controlled trial and was thus considered to have a high risk of bias, whereas the remaining 4 studies did not mention blinding and were therefore considered to have an unclear risk of bias.

#### 3.2.5. Incomplete outcome data and selective reporting

One study experienced participant dropout; however, due to the balanced reasons for dropout and the final number of participants, it was deemed to have a low risk of bias, and none of the other 4 studies had any dropouts. Additionally, there was no evidence of selective reporting in any of the RCTs, which indicated a low risk of bias.

#### 3.2.6. Other potential bias

The presence of other biases was not observed in the 5 RCTs; therefore, the risks of other biases in the RCTs was low.

### 3.3. Primary outcomes

#### 3.3.1. Effective rate

**Figure 4.**
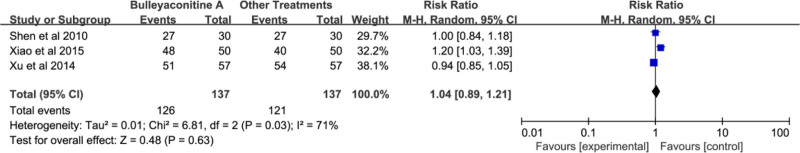
Forest plots of bulleyaconitine A and other treatments’ efficiency.

**Figure 5.**
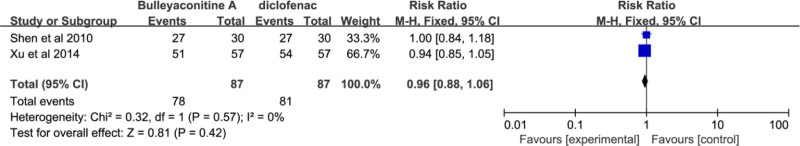
Forest plots comparing the efficiency of bulleyaconitine A and diclofenac.

The effective rates were reported in 3^[9–11]^ out of the 5 RCT studies, with a total of 274 patients involved; among them, Shen et al^[9]^ and Xu et al^[11]^ used diclofenac as the control measure, and Xiao et al^[10]^ opted for the Chinese medicine Shuangbai San. The study groups showed statistical heterogeneity (*P* = .03, *I*^2^ = 71%). We used a random-effects model for the meta-analysis. The results indicated that the efficacy of BLA in OA treatment was comparable to that of conventional therapy, with no statistical significance observed (RR = 1.04, 95% CI: 0.89–1.21, *P* = .63; Fig. 4). In the control measures for the diclofenac subgroup, we found that BLA and diclofenac had similar effects in the treatment of OA, and there was no statistically significant difference (RR = 0.96, 95% CI: 0.88–1.06, *P* = .42; Fig. 5).

#### 3.3.2. VAS score

**Figure 6.**
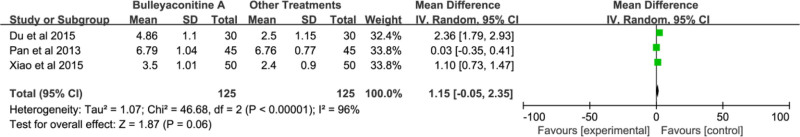
Forest plot of VAS scores at week 4 for bulleyaconitine A and other treatments. VAS = visual analogue scale.

**Figure 7.**
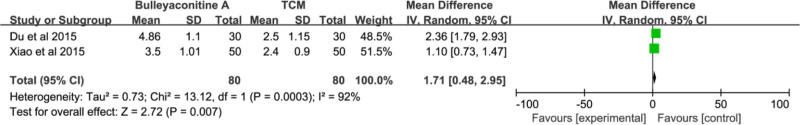
Forest plot of VAS scores at week 4 for bulleyaconitine A and TCM. TCM = traditional Chinese medicine, VAS = visual analogue scale.

The VAS scores were performed at week 4 in a total of 3 studies,^[7,8,10]^ involving 250 patients, and the study groups showed statistical heterogeneity (*P* < .1, *I*^2^ = 96%). We used a random-effects model for the meta-analysis. The study showed that the VAS score of the experimental group was similar to that of the control group at week 4 and was not statistically significant (MD = 1.15, 95% CI: −0.05 to 2.35, *P* = .06; Fig. 6). However, compared with traditional Chinese medicine, the BLA group had a significantly lower VAS score, and the difference was statistically significant (MD = 1.71, 95% CI: 0.48–2.95, *P* < .05; Fig. 7).

#### 3.3.3. Knee function score

**Figure 8.**
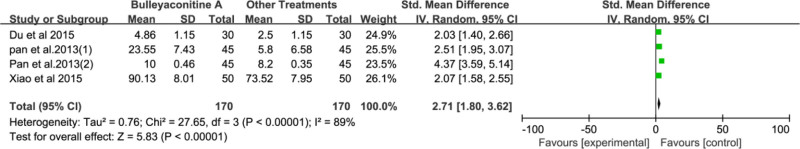
Forest plot of knee function scores at week 4 for bulleyaconitine A and other treatments.

Three studies assessed knee function scores at week 4,^[7,8,10]^ involving 250 patients, and found variations in the choice of joint function rating scales among the study groups. Pan et al^[8]^ used Lenquesne and Lysholm scores to evaluate joint function, Xiao et al^[10]^ employed the WOMAC score for joint function evaluation, and Du et al^[7]^ selected a quantitative scale of traditional Chinese medicine syndrome grading for joint function assessment. There was significant heterogeneity among the study groups (*P* < .1, *I*^2^ = 89%), thus a random-effects model was employed for data analysis. The results showed that in the 4th week, the improvement of joint function in the experimental group was significantly higher than that in the control group, which was 2.71 times of the control group, and the difference was statistically significant (SMD = 2.71, 95% CI: 1.80–3.62, *P* < .05; Fig. 8).

#### 3.3.4. Adverse events

**Figure 9.**
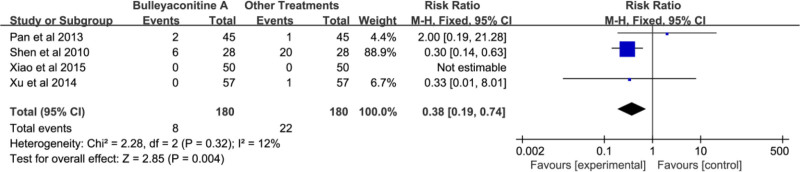
Forest plot of adverse effects of bulleyaconitine A and other treatments.

**Figure 10.**
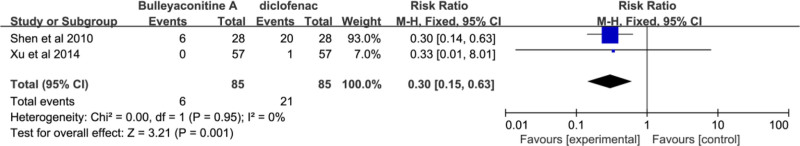
Forest plots of adverse reactions to bulleyaconitine A and diclofenac.

Four studies reported adverse events.^[8–11]^ including 360 patients; the experimental group had 8 adverse events, while the control group had 22. There was no heterogeneity among the study groups (*P* = .32, *I*^2^ = 12%). The results indicated a significantly lower incidence of adverse events in the experimental group compared to the control group, with a statistically significant difference (RR = 0.38, 95% CI: 0.19–0.74, *P* < .05; Fig. 9). Compared with the diclofenac subgroup, the BLA group had a significantly lower incidence of adverse events than the control group, and the difference was statistically significant (RR = 0.30, 95% CI: 0.15–0.63, *P* < .05; Fig. 10).

### 3.4. Publication bias

Owing to the limited number of studies included for each outcome measure, a publication bias test was not conducted in this study.

## 4. Discussion

The study included 5 RCTs with a total of 424 participants. In this systematic review and meta-analysis, we found that: Compared with NSAIDs, BLA had a similar response rate to NSAIDs but a lower incidence of adverse events. Compared with the traditional Chinese medicine, BLA can effectively reduce joint pain (decrease the VAS score), improve joint function (decrease the WOMAC score and knee pain quantitative score), and the incidence of adverse events and was equal to that of traditional Chinese medicine. Compared with hyaluronic acid, Pan Guoxing reported that, the combination of BLA and hyaluronic acid in the treatment of OA can significantly reduce joint pain (decrease VAS score) and improve joint function (decrease Lenquesne and Lysholm score). In addition, the combination therapy was superior to hyaluronic acid alone. After 6 months of follow-up, it was found that the recurrence rate in the combined treatment group was lower than that in the control group.^[8]^ BLA is superior to diclofenac sodium, hyaluronic acid, and traditional Chinese medicine in improving joint function, and this may need to be confirmed by more RCTs, and the mechanism needs further research in pharmacology.

For a long time, plant medicine in the treatment of OA has attached great importance to by the researchers. Bulleyaconitine A, a diterpenoid alkaloid extracted from Aconitum species, is a modern phytomedicine. Compared with NSAIDs, it has obvious anti-inflammatory and analgesic effects, and has higher security to gastrointestinal mucosa,^[12]^ which indicates that it may become a substitute for NSAIDs. A large number of pharmacological studies have also revealed the mechanism of BLA. The studies found that, BLA can inhibit the release of prostaglandin E in inflammatory exudates,^[13]^ reduce the level of prostaglandin E2 in serum,^[14]^ and reduce the activation and sensitization of nociceptor. It also reduces the expression of the transient receptor potential vanilloid subtype 1 channel in peripheral nerve axons, which is closely related to nociceptor activation, and relists pain.^[15]^ BLA can also regulate the expression of dynorphin A in the spinal cord and brain, increase the content of β-endorphin in serum, increase the pain threshold,^[16]^ and inhibit the C-fiber synapse-mediated long-term potentiation in the spinal transmission pathway^[17]^ to reduce pain, as well as inhibit the expression of monocyte chemotactic protein 1 and complement activation products C3a and C5a, indirectly inhibiting the chemotaxis of macrophages and reducing the inflammatory response.^[18]^ It can also inhibit the nuclear factor-kappa binding signaling pathway, inhibit the formation of osteoclasts derived from monocytes/macrophages, and promote the generation of osteoblasts, promote bone reconstruction and repair, and maintain bone homeostasis.^[19]^ Because BLA has multiple targets, it avoids the potential dangers of gastrointestinal, cardiovascular, and renal adverse reactions as well as drug dependence associated with NSAIDs and opioids, and can be used for patients who are not candidates for nonsteroidal anti-inflammatory drugs or opioids, and can be used as a long-term drug for chronic pain such as OA, rheumatoid arthritis and cancer pain, and has extensive application prospects in the clinic.

Since the mechanism of action of bulleyaconitine A is different from that of NSAIDs, whether the combination of these 2 drugs can reduce the dose and increase the efficacy while reducing the adverse effects of the drug requires long-term large-sample observation. And which drug combination of BLA and can exert the greatest efficacy still requires further exploration. Moreover, whether there are differences in the efficacy of BLA in other different ethnic groups needs further verification.

This study has the following limitations: the number of included studies was insufficient and the sample size was inadequate; the inclusion and exclusion criteria, treatment measures, and joint function scoring scales vary across studies, leading to clinical heterogeneity that may impact the analysis results; there is a lack of research on the efficacy of BLA in other formulations (such as injection and transdermal patch, etc) for the treatment of OA; due to the short duration of drug use and the lack of long-term follow-up, it is impossible to accurately evaluate long-term adverse reactions and drug effects.

## 5. Conclusion

After conducting a comprehensive analysis of the current relevant studies, we have concluded that BLA is effective in the treatment of OA with few adverse reactions. However, further validation of these findings is warranted through high-quality, large-scale, and multi-center clinical trials. Despite the limitations inherent in this study, it may serve as a valuable reference for the clinical application of BLA.

## Acknowledgments

Many thanks to the hard work of colleagues Qiang Lei and Li Li in the discussion of the significance and detailed design of the study question.

## Author contributions

**Conceptualization:** Min Xiao.

**Data curation:** Min Xiao, Xia-han Huang, Fang-lan Ma.

**Formal analysis:** Min Xiao, Xia-han Huang.

**Investigation:** Fang-lan Ma, Xia-han Huang.

**Methodology:** Min Xiao.

**Software:** Min Xiao, Xia-han Huang.

**Supervision:** Fang-lan Ma.

**Writing – original draft:** Min Xiao.

**Writing – review & editing:** Xia-han Huang.

## Supplementary Material


