# Supplementary material for: Efficacy and safety of bulleyaconitine A in the treatment of osteoarthritis: A systematic review and meta-analysis
Source: Medicine (Baltimore). 2025 Sep 12;104(37):e44389. doi: 10.1097/MD.0000000000044389 (PMC12440454; doi:10.1097/MD.0000000000044389)
Supplement: Supplementary file 1 [file medi-104-e44389-s001.doc]

**Appendix A**

1. **Appendix A1.**

1. **Appendix A2.**

1. **Appendix A3.**

1. **Appendix A4.**

1. **Appendix A5.**

1. **Appendix A6.**
